# Colchicine protects against the development of experimental abdominal aortic aneurysm

**DOI:** 10.1042/CS20230499

**Published:** 2023-10-03

**Authors:** Yi Zhao, Qi-Rui Shen, Yu-Xin Chen, Yu Shi, Wen-Bing Wu, Qiao Li, Dong-Jie Li, Fu-Ming Shen, Hui Fu

**Affiliations:** 1 Department of Pharmacy, Shanghai Tenth People’s Hospital, Tongji University School of Medicine, Shanghai, China; 2 Department of Pharmacy, Shanghai Skin Disease Hospital, Tongji University School of Medicine, Shanghai, China; 3 Department of Pharmacology, School of Pharmacy, Second Military Medical University/ Naval Medical University, Shanghai, China; 4 School of Pharmacy, Nanjing Medical University, Nanjing, China

**Keywords:** abdominal aortic aneurysm, Colchicine, Immune cells, inflammation, vascular smooth muscle

## Abstract

Abdominal aortic aneurysm (AAA) is characterized by at least 1.5-fold enlargement of the infrarenal aorta, a ruptured AAA is life-threatening. Colchicine is a medicine used to treat gout and familial Mediterranean fever, and recently, it was approved to reduce the risk of cardiovascular events in adult patients with established atherosclerotic disease. With an AAA mice model created by treatment with porcine pancreatic elastase (PPE) and β-aminopropionitrile (BAPN), this work was designed to explore whether colchicine could protect against the development of AAA. Here, we showed that colchicine could limit AAA formation, as evidenced by the decreased total aortic weight per body weight, AAA incidence, maximal abdominal aortic diameter and collagen deposition. We also found that colchicine could prevent the phenotypic switching of vascular smooth muscle cells from a contractile to synthetic state during AAA. In addition, it was demonstrated that colchicine was able to reduce vascular inflammation, oxidative stress, cell pyroptosis and immune cells infiltration to the aortic wall in the AAA mice model. Finally, it was proved that the protective action of colchicine against AAA formation was mainly mediated by preventing immune cells infiltration to the aortic wall. In summary, our findings demonstrated that colchicine could protect against the development of experimental AAA, providing a potential therapeutic strategy for AAA intervention in the clinic.

## Introduction

Abdominal aortic aneurysm (AAA) is defined as the dilatation of infrarenal aorta, of which the diameter is 1.5-fold larger than normal [1–3]. AAA is a degenerative disease due to the progressive destruction of aortic wall integrity, a ruptured AAA causes a high mortality (65–85%) in patients [4]. The pathological changes of AAA include elastin disruption, collagen deposition, degradation of extracellular matrix, loss of normal vascular smooth muscle cells (VSMCs), switching of VSMCs phenotype, enhanced inflammation and excessive oxidative stress, etc. [1,5–8]. Currently, endovascular aortic repair or open aortic repair is the only effective treatment for AAA [4,9]. However, only 10% of all patients are eligible for surgery [5]. Up to now, no proven pharmacological treatment is available to limit AAA formation and progression. Thus, testing already approved drugs and repurposing for AAA treatment deserved to study.

Colchicine is a medicine used to treat gout and familial Mediterranean fever [10,11]. The mechanism by which colchicine exerts its beneficial effects in patients with gout is to disrupt cytoskeletal functions through inhibiting β-tubulin polymerization into microtubules, and thereby prevents the activation, degranulation and migration of neutrophils, which is believed to be the pharmacological action of colchicine in control of gout symptoms [11–13]. However, the mechanism of colchicine for treatment of familial Mediterranean fever is still unclear. Evidences show that colchicine could disrupt the assembly of inflammasome in neutrophils and monocytes, and consequently inhibit interleukin-1β activating [14]. Recently, colchicine was proposed as a potentially pharmacological agent in cardiovascular diseases depending on its anti-inflammatory property [10,12]. Studies showed that colchicine treatment could reduce the risk of cardiovascular events including myocardial infarction, chronic coronary disease, recurrent pericarditis and atrial fibrillation [10,12,15–17]. Recently, it was found that colchicine exerted anti-atherosclerotic and plaque-stabilizing effects by inhibition of foam cell formation and inflammation in murine atherosclerosis [18], and more recently (in June 2023), low-dose colchicine (0.5 mg/d orally) was approved to reduce the risk of cardiovascular events in adult patients with established atherosclerotic disease or with multiple risk factors for cardiovascular disease by the U.S. Food and Drug Administration [19].

In 2022, James et al. reported that colchicine did not reduce AAA growth in a mice model with administration of colchicine (0.2 mg/kg/d via gavage) beginning from day 21 after the induction of AAA for a subsequently consecutive 69 days with the purpose to explore the therapeutic effects of colchicine on an established AAA mice model [20]. Given the facts that AAA is life threatening and associated with vascular walls’ inflammation, and colchicine could prevent neutrophils migration and interleukin-1β activation, we hypothesized that colchicine could protect against the development of AAA at the early stage. In this work, with an AAA mice model induced by porcine pancreatic elastase (PPE) and β-aminopropionitrile (BAPN), colchicine was given intraperitoneally once a day for 2 weeks throughout the whole experimental procedure. The effects of colchicine on AAA formation, inflammation and immune cells infiltration were assessed.

## Methods

### Mice and AAA model

Eight-week-old male C57BL/6 mice were purchased from Shanghai SLAC Laboratory Animal Co. Ltd (Shanghai, China). The mice were housed in standard conditions with a relative humidity of approximately 50%, a constant temperature of 22–25°C, a 12-h light/12-h dark cycle, and free access to food and water in the animal house of Tongji University. The animals’ maintenance and the experimental procedures were in compliance with the Guide for Care and Use of Experimental Animals approved by the Animal Center, Tongji University. All of the animal experiments were conducted in the laboratory of Tongji University.

Healthy 8-week-old male C57BL/6 mice were used to induce AAA formation by PPE and BAPN [21,22]. Briefly, mice were given pentobarbital sodium (50 mg/kg, i.p.) for anesthesia and positioned in a supine posture on an animal surgical operating table. About 1.5 cm midline incision was made in the mouse abdominal wall, the abdominal aorta from infrarenal aorta to bifurcation of the aorta was slightly isolated. The isolated aorta was then wrapped circumferentially by blotting (bibulous) paper about 1 mm wide and 4 mm long soaked with 4 U PPE for 40 min, the control was treated with saline. Then, the blotting paper was removed, and the abdomen was sutured. After surgery, the mice were given 0.4% BAPN drinking water for 2 weeks. Colchicine (0.1 mg/kg, i.p.) was injected immediately after operation, and was given once a day for a total of 2 weeks. After that, mice were euthanized by cervical dislocation under anesthesia (pentobarbital sodium, 150 mg/kg, i.p.), and the entire abdominal aorta was dissected.

### Morphological analysis and immunohistochemistry staining

The dissected entire aorta was pictured on a black background to display the size of the abdominal aortic aneurysm. After that, the abdominal aortic aneurysm was immersed and fixed in 4% polyformaldehyde, embedded in paraffin, and then sectioned and stained. Hematoxylin–eosin (HE) staining combined with a software (ImageJ) was used to measure the diameter of the abdominal aorta. Masson’s trichrome staining was used to determine the deposition of collagen fibers, while Verhoeff-van Gieson (VVG) staining was used to assess the integrity of elastic fibers. The scoring criteria for elastic fibers are as follows: score 1, the elastic laminae maintained intact and without degradation; score 2, certain breaks existed in the elastic laminae; score 3, severe elastin fragmentations appeared in aorta; score 4, severe elastin degradation occurred in aorta.

### Western blot

Proteins were extracted from mice abdominal aortic tissues and were subjected to Western blot analysis after quantification using a BCA assay kit (Thermo Scientific, U.S.A.). After incubating with primary antibodies at 4°C overnight, the samples were exposed to secondary antibodies for 30 min at room temperature. Protein expressions were detected using the Odyssey infrared imaging system from LICOR (U.S.A.), and the data were analyzed by ImageJ and normalized to the corresponding β-actin. Primary antibodies of α-SMA (14395-1-AP), PCNA (10205-2-AP), MMP2 (10373-2-AP), MMP9 (10375-2-AP), NOX2 (19013-1-AP), NOX4 (14347-1-AP), IL-18 (10663-1-AP) and Caspase-1 (22915-1-AP) were purchased from Proteintech (U.S.A.). IL-1β (sc-515598), TNF-α (sc-52746), OPN (sc-21742), F4/80 (sc-377009), Ly6G (sc-53515) and CD3 (sc-20047) antibodies were purchased from Santa Cruz (U.S.A.). ASC (#67824) antibody was purchased from Cell Signaling Technology (U.S.A.). GSDMD (ab209845) antibody was purchased from Abcam (U.K.). NLRP3 (AF2155) and β-actin (AF2815) antibodies were purchased from Beyotime (China).

### Immunofluorescence staining

Samples of mice abdominal aortic aneurysms were immersed and fixed in 4% polyformaldehyde, dehydrated in 20% sucrose, embedded in OCT and sliced into 6 μm sections. The sections were then blocked with 20% goat serum for 3 h at room temperature and subsequently incubated with primary antibodies at 4°C overnight. The sections were further incubated with Alexa Fluor-labeled secondary antibodies for 30 min at room temperature. Nuclei were stained with DAPI for 15 min, and the immunofluorescence images were captured using the microscopy (BX51, Olympus, Tokyo, Japan). Primary antibodies were the same as those listed in the Western blot part, IgG (A0216) antibody was purchased from Beyotime (China). The efficacy of the above antibodies was validated and detailed positive/negative controls were provided in the Supplementary Figure S1.

### AAA lesion cell flow cytometry

AAA lesion cells were isolated with the aorta dissociation enzyme stock solution (ADES: 1300 U/ml collagenase type I, 60 U/ml hyaluronidase type 1-s, and 60 U/ml DNase I, in 2 ml of HBSS). All enzymes were purchased from Worthington Biochemical (U.S.A.). The pre-prepared enzyme solution was kept at 4°C. Rinsing the aorta in HBSS and then removed it into 2 ml of ADES. For better digestion, the aortas were sliced into little pieces and were incubated with the ADES for 1 h at 37°C. Then, stop digestion with HBSS containing 10% fetal bovine serum (FBS). By passing the digested aorta solution through a 70 μm cell strainer (BIOFIL CAT: CSS013070), AAA single cell suspensions were prepared. Cells were centrifuged (1,200 rpm, 10 min, 4°C) and resuspended in 500 μl of HBSS. To measure macrophages, neutrophils and T cells, AAA single cell suspensions were stained with cell viability dye (Cat#79997) and cell surface marker antibodies. CD45 (Cat#103139), CD163 (Cat#155319), CD86 (Cat#105039), CD3 (Cat#100204) and CD8 (Cat#100708) were purchased from Biolegend (US.A.). CD11b (Cat#564454), F4/80 (Cat#565787), Ly6G (Cat#562737) and CD4 (Cat#553051) were purchased from BD Pharmingen (U.S.A.). After staining for 30 min at 4°C in dark, cells were washed in HBSS and run for data acquisition with a Beckman CytoFLEX (U.S.A.). Data were analyzed by the FlowJo software.

### Statistical analysis

Data were expressed as mean ± SEM. One-way ANOVA for four groups and unpaired *t*-test for two groups were used for statistical analysis. AAA incidence analysis was conducted using Fisher’s exact test. And the one-tailed Wilcoxon test was used to evaluate the levels of elastin degradation among different groups. A value of *P*<0.05 was considered as statistically significance. GraphPad Prism Software 8.0 was used to conduct all of the statistical analyses.

## Results

### Colchicine limited AAA formation in mice

**Figure 1:**
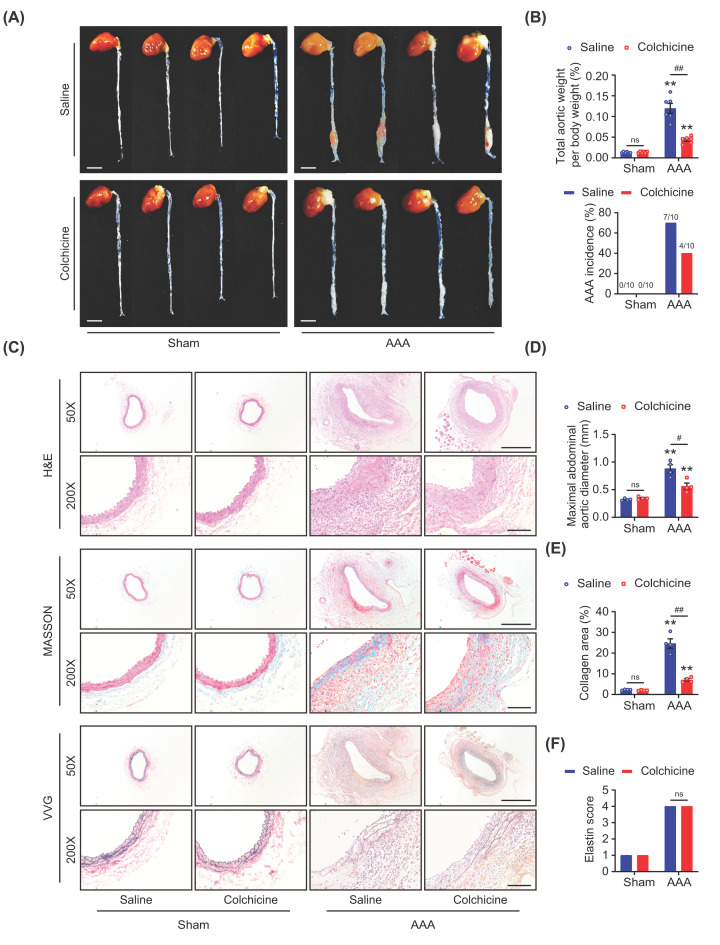
Colchicine limited AAA formation in mice Male C57BL/6 mice were treated with porcine pancreatic elastase (PPE, 4U) incubation and 0.4% β-aminopropionitrile (BAPN) in drinking water for 2 weeks to induce abdominal aortic aneurysm (AAA). Colchicine (0.1 mg/kg/d, *i.p*.) was injected after operation for 2 weeks. (**A**) Representative images of mice aortas, scale bar = 4 mm, *n* = 4 per group. (**B**) Total aortic weight per body weight, *n* = 6 per group; incidence of AAA, *n* = 10 per group. (**C**) Representative images of HE, Masson, VVG staining, scale bar = 500 μm (up) or 100 μm (down). Colchicine treatment reduced maximal abdominal aortic diameter (**D**) and collagen deposition (**E**), and with no effects on elastin score evaluation (**F**) in AAA groups, *n* = 4 per group. Data were shown as mean ± SEM, **P<0.01 vs. Sham; ^#^*P*<0.05, ^##^*P*<0.01 vs. AAA + Saline; ns, no significance.

AAA was induced by PPE incubation combined with BAPN in drinking water [21–24]. Two weeks later, it was found that the size of AAA was reduced (Figure 1A), and both the total aortic weight per body weight and the AAA incidence (4/10 vs. 7/10) were lower in the colchicine treated mice when compared with the control (Figure 1B). No significant changes of these observed indexes were found between the saline and colchicine treated group without combination use of PPE and BAPN (Figure 1A,B). After AAA induction, obvious aortic lumen dilation, collagen deposition, elastin depletion, as well as disruption and thickness in aortic medial layer and adventitia were observed by histological examination. Importantly, treatment with colchicine partially prevented these changes (Figure 1C). Statistical analysis showed that either the maximal abdominal aortic diameter or the collagen deposition in AAA tissues was significantly decreased by colchicine administration as compared with the control (Figure 1D,E). Though it seems that colchicine administration preserved part of the elastin fibers, elastin score evaluation showed no significant difference between two AAA groups (Figure 1F). These suggested that colchicine administration could limit AAA formation in mice.

### Colchicine prevented phenotypic switching of VSMCs in AAA mice

**Figure 2:**
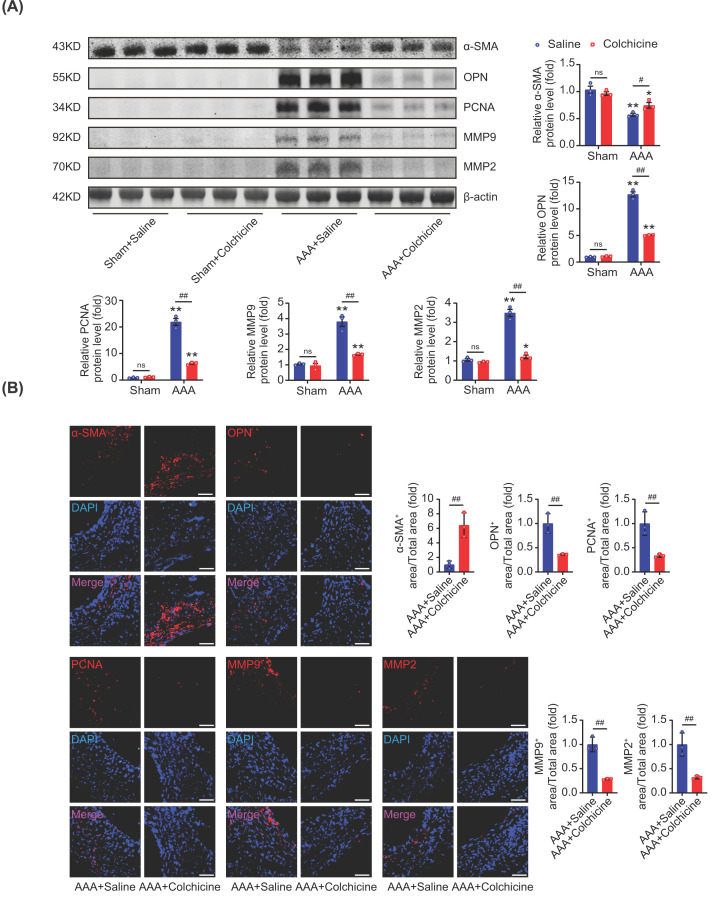
Colchicine prevented phenotypic switching of VSMCs in AAA mice Male C57BL/6 mice were treated with porcine pancreatic elastase (PPE, 4U) incubation and 0.4% β-aminopropionitrile (BAPN) in drinking water for 2 weeks to induce abdominal aortic aneurysm (AAA). Colchicine (0.1 mg/kg/d, i.p.) was injected after operation for 2 weeks. (**A**) Colchicine treatment up-regulated the expression of α-SMA and down-regulated OPN, PCNA, MMP9 and MMP2 in AAA tissues. (**B**) Representative images of immunofluorescence staining of α-SMA (red), OPN (red), PCNA (red), MMP9 (red) and MMP2 (red) in AAA tissues; nuclei were counterstained with DAPI (blue), scale bar = 100 μm. *n* = 3 per group, data were shown as mean ± SEM, *P<0.05, **P<0.01 vs. Sham; ^#^*P*<0.05, ^##^*P*<0.01 vs. AAA + Saline; ns, no significance.

During the progression of AAA, VSMCs could switch from the contractile phenotype to the synthetic phenotype, which was considered to be a critical characteristic of AAA [6–8]. Meanwhile, abnormal cell proliferation and excessive degradation of extracellular matrix (ECM) were also believed to be pivotal during the development of AAA [23–25]. Western blot analyses and immunofluorescence staining showed that colchicine treatment increased the expression of α-SMA (a marker of VSMCs contractile phenotype) and suppressed the expression of OPN (a marker of VSMCs synthetic phenotype) after AAA induction (Figure 2A,B). It was also found that the level of PCNA (a marker of cell proliferation) was significantly reduced by colchicine treatment. In addition, both matrix metalloproteinase-9 (MMP9) and matrix metalloproteinase-2 (MMP2), which was able to degrade ECM, were also inhibited by colchicine (Figure 2A,B). These together suggested that colchicine treatment could prevent phenotypic switching of VSMCs and ECM degradation in AAA mice.

### Colchicine inhibited vascular inflammation and oxidative stress in AAA mice

**Figure 3:**
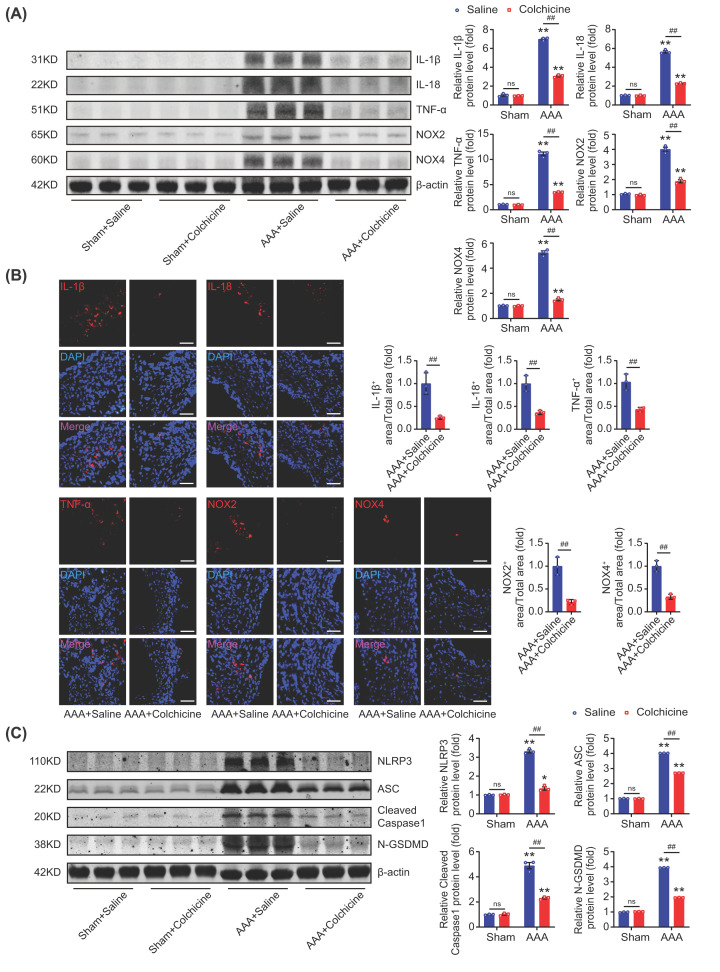
Colchicine inhibited vascular inflammation and oxidative stress in AAA mice Male C57BL/6 mice were treated with porcine pancreatic elastase (PPE, 4U) incubation and 0.4% β-aminopropionitrile (BAPN) in drinking water for 2 weeks to induce abdominal aortic aneurysm (AAA). Colchicine (0.1 mg/kg/d, i.p.) was injected after operation for 2 weeks. (**A**) Colchicine treatment down-regulated the expressions of IL-1β, IL-18, TNF-α, NOX2 and NOX4 in AAA tissues. (**B**) Representative images of immunofluorescence staining of IL-1β (red), IL-18 (red), TNF-α (red), NOX2 (red) and NOX4 (red) in AAA tissues; nuclei were counterstained with DAPI (blue), scale bar = 100 μm. (**C**) Colchicine treatment down-regulated the protein expressions of NLRP3, ASC, cleaved caspase-1 and N-GSDMD in AAA tissues. *n* = 3 per group, data were shown as mean ± SEM, *P<0.05, **P<0.01 vs. Sham; ^##^*P*<0.01 vs. AAA + Saline; ns, no significance.

Inflammation and excessive oxidative stress in the aortic wall were the major pathological process in AAA [6,24,26–28]. IL-1β, IL-18 and TNF-α were the typical inflammatory hallmarks in AAA [29–32], and NOX2 and NOX4 were the mostly reported members of the NADPH oxidase (NOX) family, and contributed the main source of reactive oxygen species (ROS) in AAA [26,33–36]. It was found that the expressions of IL-1β, IL-18 and TNF-α were significantly inhibited in colchicine treated AAA mice when compared with the saline treated AAA ones. Similar results were found for NOX2 and NOX4 (Figure 3A,B). NLRP3/caspase-1/GSDMD-dependent cell pyroptosis was believed to be involved in the formation and progression of AAA, which subsequently led to the release of IL-1β and IL-18, etc. [3,37]. In this work, it was found that the expressions of NLRP3, ASC, cleaved caspase-1 and N-GSDMD were significantly inhibited in colchicine treated AAA mice than that in saline treated AAA mice (Figure 3C). These collectively indicated that colchicine could inhibit vascular inflammation and oxidative stress in AAA mice.

### Colchicine inhibited immune cells infiltration to AAA lesions

**Figure 4:**
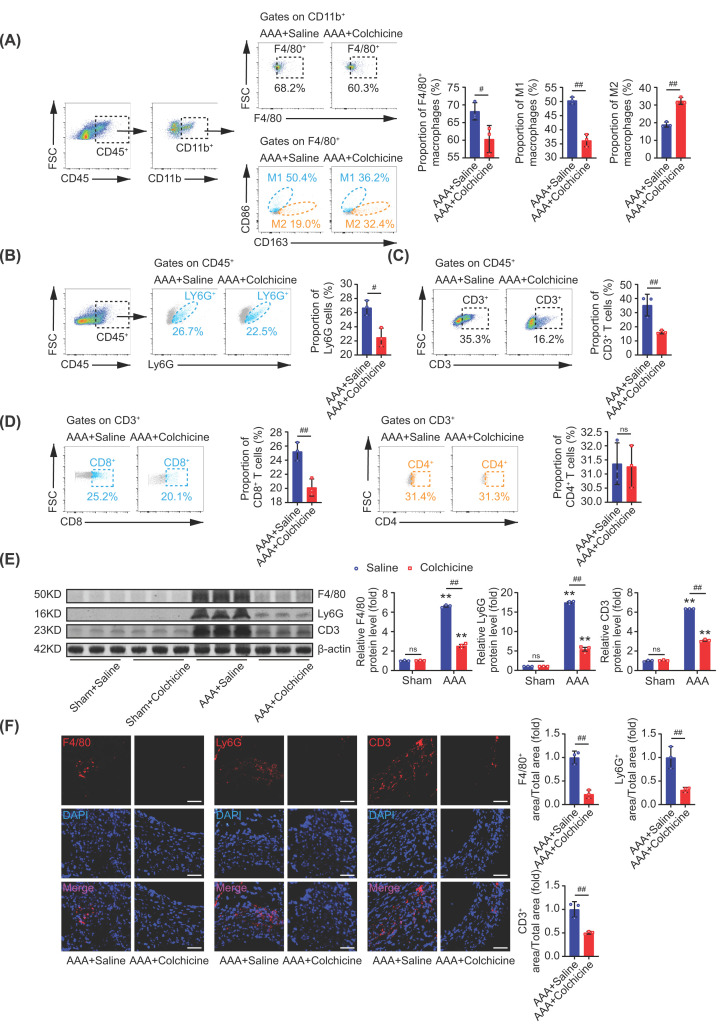
Colchicine inhibited immune cells infiltration to AAA lesions Male C57BL/6 mice were treated with porcine pancreatic elastase (PPE, 4U) incubation and 0.4% β-aminopropionitrile (BAPN) in drinking water for 2 weeks to induce abdominal aortic aneurysm (AAA). Colchicine (0.1 mg/kg/d, *i.p*.) was injected after operation for 2 weeks. By flow cytometric sorting, the aortic immune cells of AAA lesion were isolated. (**A**) Colchicine treatment reduced the percentage of total macrophages (CD45^+^CD11b^+^F4/80^+^) and M1 macrophages (CD45^+^CD11b^+^F4/80^+^CD86^+^), while increased the proportion of M2 macrophages (CD45^+^CD11b^+^F4/80^+^CD163^+^). (**B–D**) Colchicine treatment reduced the percentage of neutrophils (CD45^+^Ly6G^+^), total T cells (CD45^+^CD3^+^) and cytotoxic T cells (CD45^+^CD3^+^CD8^+^), no significant differences were detected for T helper cells (CD45^+^CD3^+^CD4^+^). (**E**) Colchicine treatment down-regulated the expressions of F4/80, Ly6G and CD3 in AAA tissues. (**F**) Representative images of immunofluorescence staining of F4/80 (red), Ly6G (red) and CD3 (red) in AAA tissues, nuclei were counterstained with DAPI (blue), scale bar = 100 μm. *n* = 3 per group, data were shown as mean ± SEM, **P<0.01 vs. Sham; ^#^*P*<0.05, ^##^*P*<0.01 vs AAA + Saline; ns, no significance.

It is well-known that colchicine was able to inhibit the migration and adhesion of leukocytes [10,12]. While a distinct characteristic of AAA is the infiltration of immune cells [23,38–40]. To further understand the effect of colchicine on AAA, we detected the infiltration of macrophages, neutrophils and lymphocytes in AAA. By flow cytometric sorting, the aortic immune cells from murine AAA tissues were isolated. Different antibodies were used to distinguish various cell populations: leukocyte (CD45), monocyte (CD11b), macrophage (F4/80), M1 macrophage (CD86), M2 macrophage (CD163), neutrophil (Ly6G), T cell (CD3), cytotoxic T cell (CD8) and T helper cell (CD4) [31,38,39,41–45]. It was demonstrated that colchicine treatment significantly reduced the percentage of total macrophages, M1 macrophages, neutrophils, total T cells and cytotoxic T cells, while increased the proportion of M2 macrophages when compared to the control after AAA induction (Figure 4A–D). No significant difference was detected for T helper cells (Figure 4D). To validate these results, we again assessed the protein expressions of F4/80, Ly6G and CD3 by Western blot and immunofluorescence staining, and found that the infiltration of macrophages, neutrophils and T cells in AAA tissues were all limited after colchicine treatment (Figure 4E,F). Taken together, these findings suggested that colchicine might inhibit immune cells’ migration, adhesion and infiltration to AAA lesions, thereby reducing inflammation during AAA formation.

### Colchicine limited AAA formation by inhibiting immune cells infiltration

**Figure 5:**
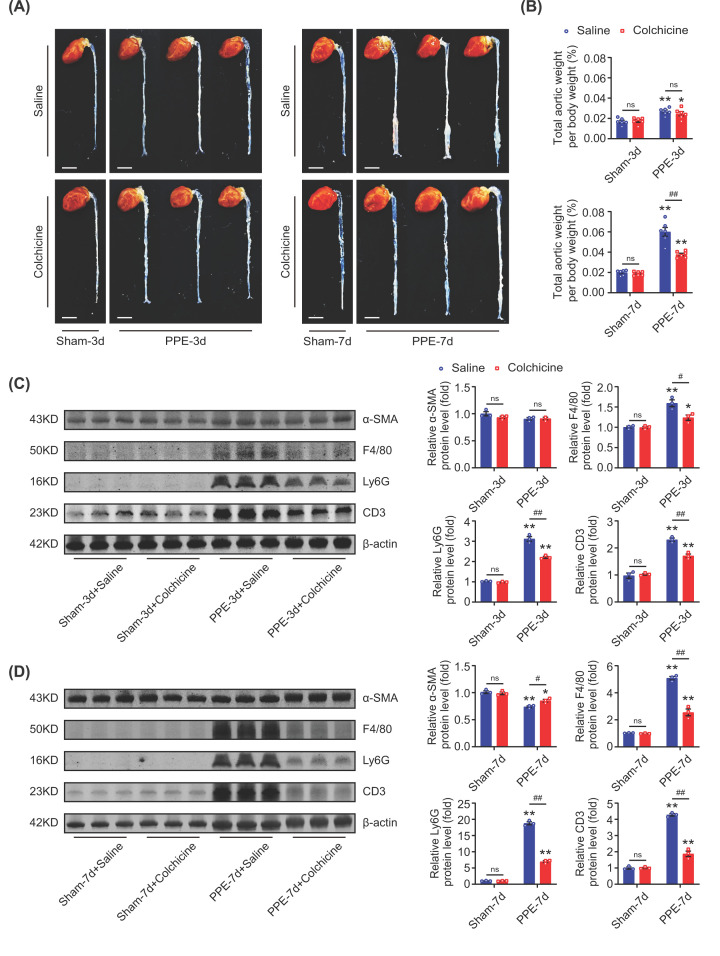
Colchicine limited AAA formation by inhibiting immune cells infiltration Male C57BL/6 mice were treated with porcine pancreatic elastase (PPE, 4U) incubation for 40 min and 0.4% β-aminopropionitrile (BAPN) in drinking water for 3 and 7 days, respectively. Colchicine (0.1 mg/kg/d, i.p.) was injected after operation for 3 and 7 days, respectively. (**A**) Representative images of mice aortas, scale bar = 4 mm, *n* = 4 per group. (**B**) Total aortic weight per body weight, *n* = 6 per group. (**C**) Colchicine treatment did not affect the expression of α-SMA, while down-regulated the expressions of F4/80, Ly6G and CD3 in abdominal aortic tissues on the third day. (**D**) Colchicine treatment slightly up-regulated the expression of α-SMA, and down-regulated the expressions of F4/80, Ly6G and CD3 in abdominal aortic tissues on the seventh day. *n* = 3 per group, data were shown as mean ± SEM, *P<0.05, **P<0.01 vs. Sham; ^#^*P*<0.05, ^##^*P*<0.01 vs. PPE + Saline; ns, no significance.

To further explore the action of colchicine on the development of AAA, we observed the visible changes of the abdominal aortas, and measured the expression of α-SMA and the infiltration of immune cells in the aortic wall at day 3 and day 7 after PPE incubation. At day 3 after PPE incubation, no visible enlargement of abdominal aortas was observed in either saline or colchicine treated mice (Figure 5A), and also the total aortic weight per body weight and protein level of α-SMA were not changed between the two groups (Figure 5B,C). However, the increased expressions of F4/80 (marker of the macrophages), Ly6G (marker of the neutrophils) and CD3 (marker of the T cells) induced by PPE were significantly inhibited by colchicine treatment (Figure 5C). At day 7, the enlargement of abdominal aortas, the increased total aortic weight per body weight, the decreased protein level of α-SMA, and the increased expressions of F4/80, Ly6G and CD3 caused by PPE were all attenuated by colchicine treatment (Figure 5A,B,D). These suggested that the protective action of colchicine against AAA formation might be dependent on preventing immune cells infiltration to the aortic wall.

## Discussion

AAA is a lethal disease and nowadays there are no proved drugs for the treatment of AAA [5,9]. One of the prominent characteristics of AAA is infiltration of immune cells and inflammation in the aortic wall [23,38,39]. Colchicine, an anti-gout drug world widely used in the clinic is able to prevent neutrophils migration and inhibit inflammation [10,12]. The main findings of this work are: colchicine could limit AAA formation, prevent phenotypic switching of VSMCs, and inhibit inflammation and immune cells infiltration to the aortic wall in a PPE-induced experimental AAA mice model.

Recently, PPE incubation combined with BAPN drinking water has become a widely used method to induce AAA [21,22]. With this experimental AAA mice model, the protective effect of colchicine on AAA was assessed. It was found that colchicine treatment alleviated the pathological changes including collagen deposition, aortic wall disruption and thickness. Furthermore, colchicine reduced maximal abdominal aortic diameter and AAA incidence. These demonstrated that colchicine injection (0.1 mg/kg, i.p.) once a day for 2 weeks throughout the experimental procedure could limit AAA formation.

Phenotype switching of VSMCs, excessive cell proliferation and degradation of ECM could aggravate the pathological vascular remodeling, destroy the integrity of aortic wall, etc*.*, and thus stimulate the pathological process of AAA [23,24]. The degradation of ECM was caused by the secretion of MMPs produced by VSMCs and inflammatory cells in AAA [2]. MMP9 and MMP2 were the most reported members of the MMP family involved in the progression of AAA [9,46]. In this work, we found that colchicine preserved the contractile type of VSMCs and reduced the synthetic type of VSMCs. Besides, colchicine treatment reduced the expressions of PCNA (a marker of cell proliferation), MMP9 and MMP2. These indicated that colchicine could prevent the phenotypic switching of VSMCs from the contractile to synthetic state, and alleviate pathological vascular remodeling during AAA formation.

Inflammation is a prominent characteristic of AAA, which was described as the accumulation of inflammatory cytokines in aortic walls [6,24]. Excessive oxidative stress also played an important role in the development of AAA [26]. Cell pyroptosis, a form of programmed cell death during inflammation, was reported to be involved in the progression of AAA [3]. We found that colchicine could decrease the vascular inflammation (determined by IL-1β, IL-18 and TNF-α), oxidative stress (determined by NOX2 and NOX4) and cell pyroptosis (determined by NLRP3, ASC, cleaved caspase-1 and N-GSDMD) in AAA mice. A well accepted pharmacological action of colchicine is inhibiting the migration and adhesion of leukocytes [10,12]. To explore whether colchicine limited AAA progression by preventing immune cells migration, AAA lesion cells were analyzed by flow cytometry. It was considered that M1 macrophages, neutrophils and cytotoxic T cells contributed to production of inflammatory cytokines [23,47–49]. While M2 macrophages was generally believed to possess anti-inflammatory action [50]. In this work, we did find that colchicine reduced the proportion of total macrophages, M1 macrophages, neutrophils, total T cells and cytotoxic T cells. While the proportion of M2 macrophages was increased. These suggested that colchicine could relieve vascular inflammation by preventing immune cells infiltration during AAA progression.

To further investigate the impact of colchicine at the early stage of AAA, we observed the visible changes of the abdominal aortas, and assessed the infiltration of immune cells in the aortic wall at day 3 and day 7 after PPE incubation. At day 3 after PPE incubation, no visible enlargement of abdominal aortas was observed in either colchicine or saline treated mice. However, the expressions of F4/80 (marker of the macrophages), Ly6G (marker of the neutrophils) and CD3 (marker of the T cells) were significantly increased when compared with the time-matched sham-operated mice, which were reduced by colchicine treatment. At day 7, the enlargement of abdominal aortas, and the increased expressions of F4/80, Ly6G and CD3 caused by PPE were all attenuated by colchicine treatment. These suggested that the protective action of colchicine against AAA formation might be dependent on preventing immune cells infiltration to the aortic wall at the beginning of AAA.

In 2022, James et al. reported that colchicine could not limit the growth of already established AAA [20]. James’ work and ours were both designed to investigate the impact of colchicine on AAA formation but focused on different stages. James’ work found that colchicine did not significantly reduce AAA growth with a 69-day administration of colchicine started from day 21 after AAA induction for the purpose to explore the therapeutic effects of colchicine on an established AAA mice model. In our work, we gave colchicine immediately from the first day of the surgery for a consecutive 14-day and found that colchicine could limit the growth of AAA with the aim of exploring the preventive effects of colchicine on AAA formation at the early stage. We postulated that the different results between James’ and ours were mainly caused by the different initiation of colchicine after AAA induction. Compared with James’ work, the relatively small sample size and the missing endpoint events (aneurysm rupture and mortality rate) were our work’s limitation.

The mechanism of action of colchicine is complicated, at least include inhibiting tubulin polymerization, neutrophil migration and leukocyte adhesion, suppressing activation of NLRP3 inflammasome and proliferation of smooth muscle cell etc. [10,12]. It is well accepted that the AAA growth is boosted by ageing, atherosclerosis and smoking etc*.*, and AAA is positively related to vascular inflammatory infiltration. Nowadays, no proven pharmacological treatment is available to limit AAA formation and progression [51]. Maybe colchicine could not limit the growth of AAA eventually, our work indicated that colchicine played a protective role at the early stage of AAA formation, and further studies are needed.

In summary, our findings demonstrated that colchicine could protect against the development of AAA at the early stage in mice. The protective effect of colchicine was related to inhibiting vascular inflammation possibly dependent on preventing immune cells infiltration. Our work provided evidence that repurposing of colchicine for AAA treatment deserved further investigation.

## Clinical perspectives

- AAA is a lethal disease and nowadays there are no proved drugs for the treatment of AAA. Colchicine, an anti-gout drug world widely used in the clinic, and recently (in June 2023) being approved to reduce the risk of cardiovascular events in adult patients by FDA, is able to prevent neutrophil migration and inhibit inflammation.
- The main findings of this work are: colchicine could limit AAA formation, prevent phenotypic switching of VSMCs, and inhibit inflammation and immune cells infiltration to the aortic wall in a mice AAA model with PPE and BAPN.
- Our work provided evidence that repurposing of colchicine for AAA treatment deserved further investigation.

## Supplementary Material

Supplementary Figures S1-S5

## Data Availability

The data that support the findings of this study are available in the supplementary material of this article and we can provide other original data upon your request.

## Abbreviations

AAA: abdominal aortic aneurysm
PPE: porcine pancreatic elastase
BAPN: β-aminopropionitrile
VSMC: vascular smooth muscle cell
HE: Hematoxylin–eosin
VVG: Verhoeff-van Gieson
ECM: extracellular matrix
α-SMA: α-smooth muscle actin
PCNA: proliferating cell nuclear antigen
MMP9: matrix metalloproteinase-9
MMP2: matrix metalloproteinase-2
ROS: reactive oxygen species
