# Supplementary material for: Colchicine protects against the development of experimental abdominal aortic aneurysm
Source: Clin Sci (Lond). 2023 Oct 3;137(19):1533–45. doi: 10.1042/CS20230499 (PMC10550771; doi:10.1042/CS20230499)
Supplement: Supplementary Figures S1-S5 [file CS-2023-0499_supp.pdf]

Supplementary Materials and Methods

Male C57BL/6 mice were treated with saline incubation for 40 minutes and normal drinking water for 2 weeks, or male C57BL/6 mice were treated with porcine pancreatic elastase (PPE, 4U) incubation for 40 minutes and 0.4% beta aminopropionitrile (BAPN) drinking water for 2 weeks to induce abdominal aortic aneurysm (AAA) formation. Colchicine (0.1 mg/kg/d, *i.p.*) was injected after operation once a day for 2 weeks, and the control was given the same volume of saline once a day for 2 weeks.

The abdominal aortas were harvested and fixed in 4% polyformaldehyde for immunofluorescence staining. Representative images were pictured, including α-SMA (red), OPN (red), PCNA (red), MMP9 (red), MMP2 (red), IL-1β (red), IL-18 (red), TNF-α (red), NOX2 (red), NOX4 (red), F4/80 (red), Ly6G (red) and CD3 (red) in abdominal aortas, nuclei were counterstained with DAPI (blue). Representative images of immunofluorescence staining of normal IgG (red) in AAA tissues were also pictured, and the nuclei were counterstained with DAPI (blue).

Supplementary Results

No significant difference was found between sham groups (saline *vs* colchicine), and almost no red fluorescence was detected with antibodies (OPN, PCNA, MMP9, MMP2, IL-1β, IL-18, TNF-α, NOX2, NOX4, F4/80, Ly6G and CD3) except for α-SMA, which could be regarded as the positive control (Supplemental Figure 1A). In addition, no significant difference was found between AAA groups (saline *vs* colchicine), and almost no red fluorescence was detected with normal IgG

(Supplemental Figure 1B). It served as a negative control.

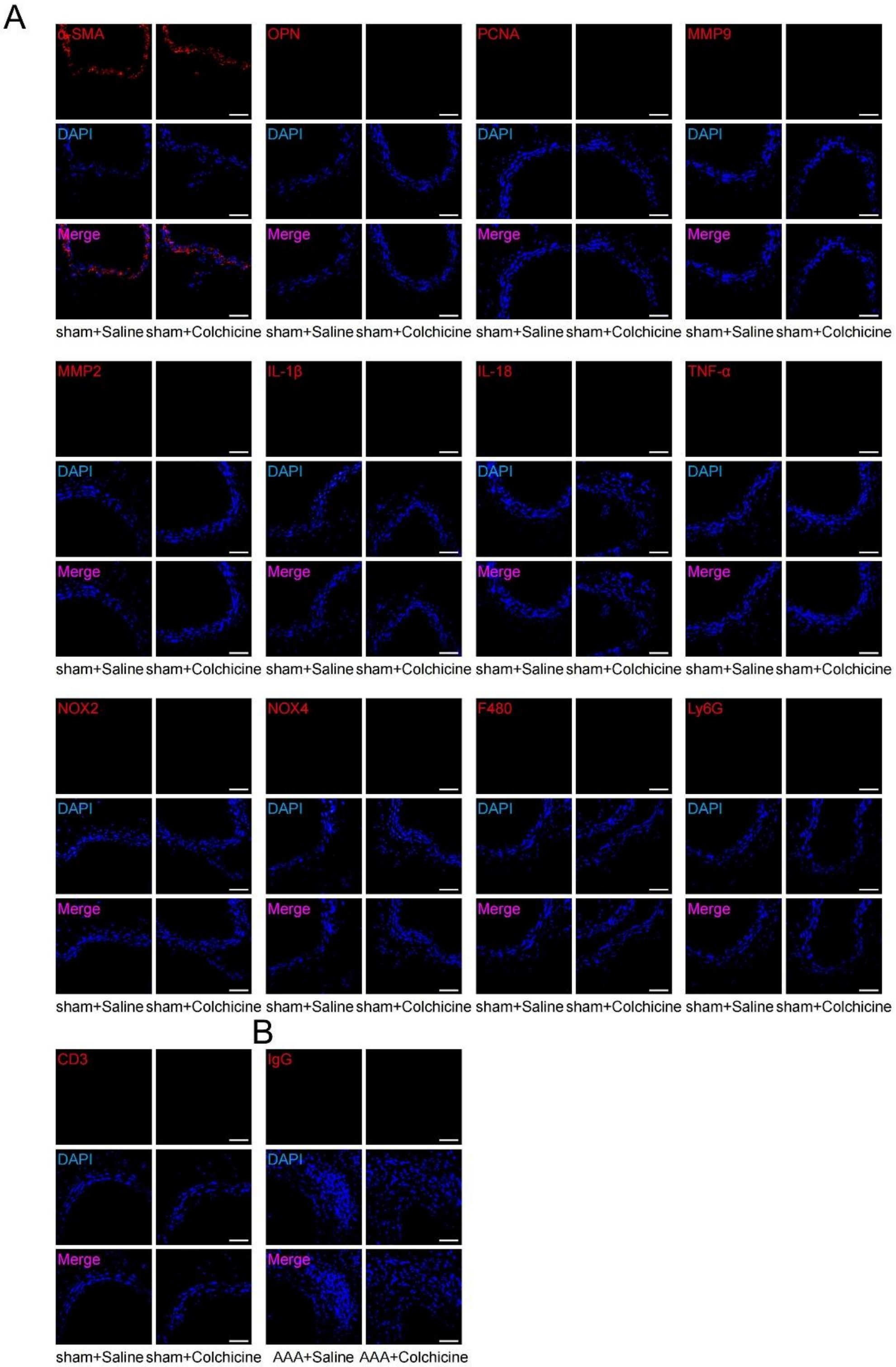

Supplemental Figure 1

Supplementary Figure 1. Representative images of positive/negative immunofluorescence staining. Male C57BL/6 mice were treated with saline incubation for 40 minutes and normal drinking water for 2 weeks, or male C57BL/6 mice were treated with porcine pancreatic elastase (PPE, 4U) incubation for 40 minutes and 0.4% beta aminopropionitrile (BAPN) drinking water for 2 weeks to induce abdominal aortic aneurysm (AAA) formation. Colchicine (0.1 mg/kg/d, i.p.) was injected after operation once a day for 2 weeks, and the control was given the same volume of saline once a day for 2 weeks. (A) Representative images of immunofluorescence staining of α-SMA (red), OPN (red), PCNA (red), MMP9 (red), MMP2 (red), IL-1β (red), IL-18 (red), TNF-α (red), NOX2 (red), NOX4 (red), F4/80 (red), Ly6G (red) and CD3 (red) in abdominal aortic tissues, nuclei were counterstained with DAPI (blue), scale bar = 100μm. (B) Representative images of immunofluorescence staining of IgG (red) in AAA tissues, nuclei were counterstained with DAPI (blue), scale bar = 100μm.

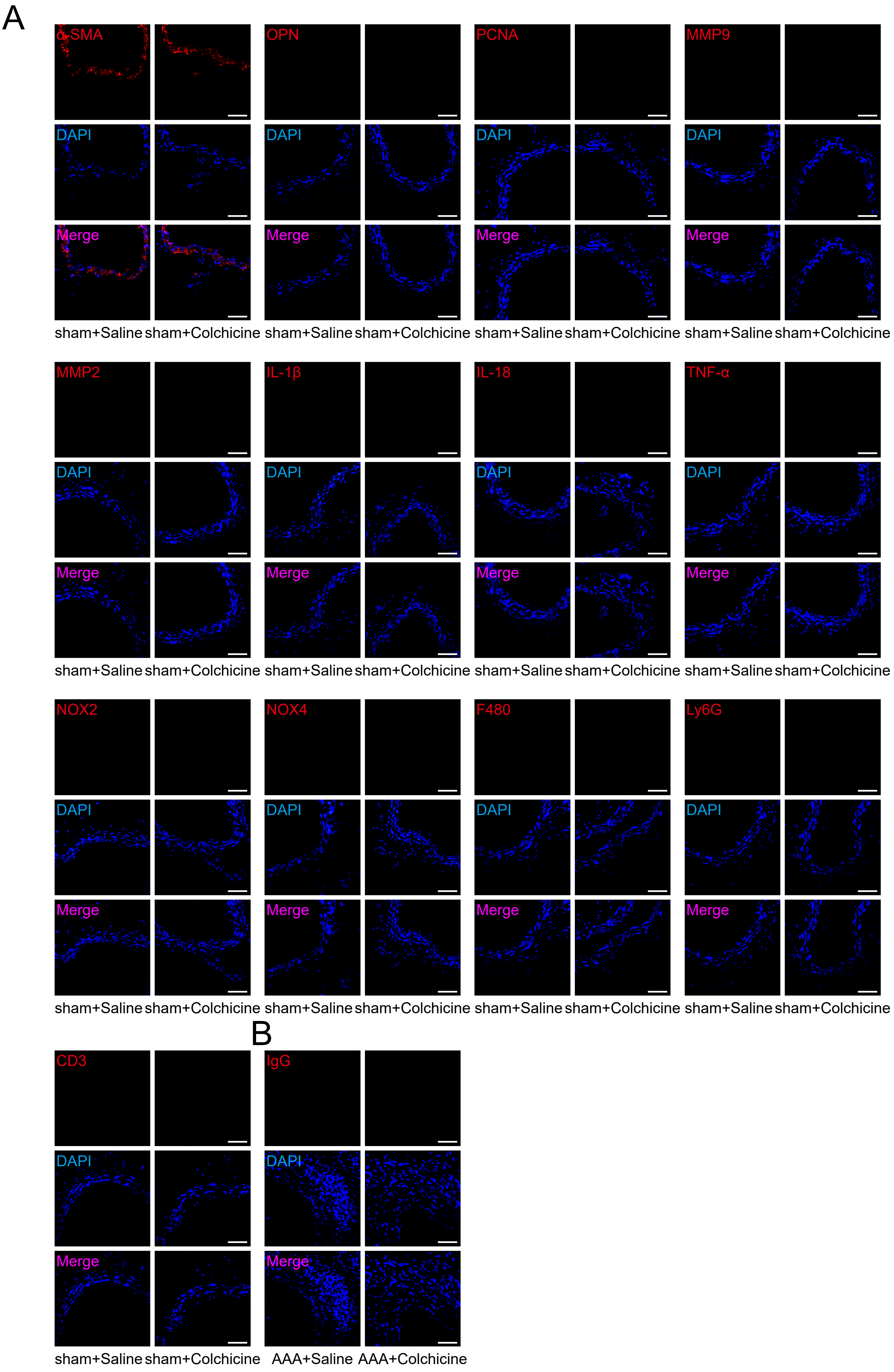

Supplemental Figure 1

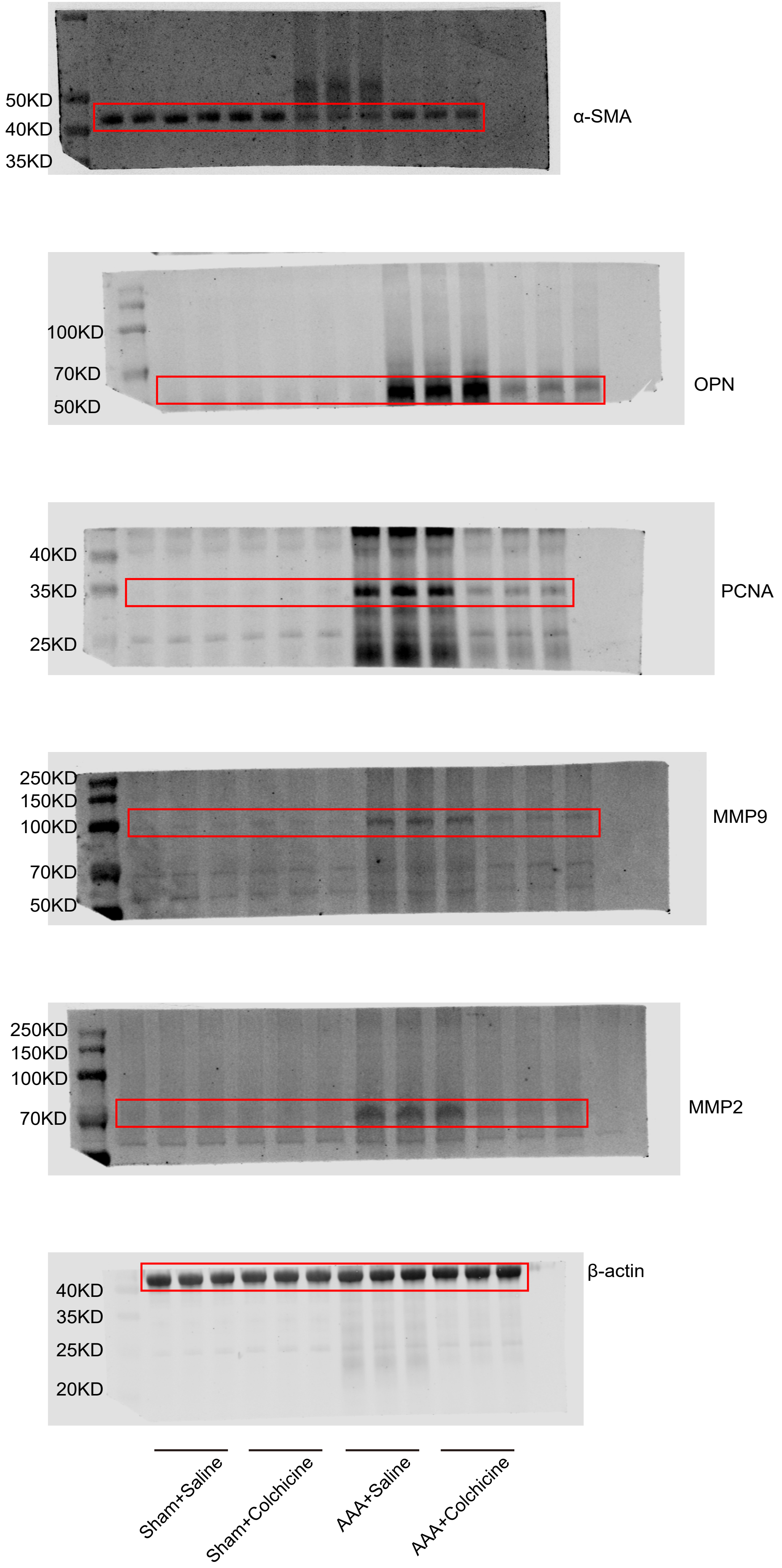

Figure 2 Supplementary material

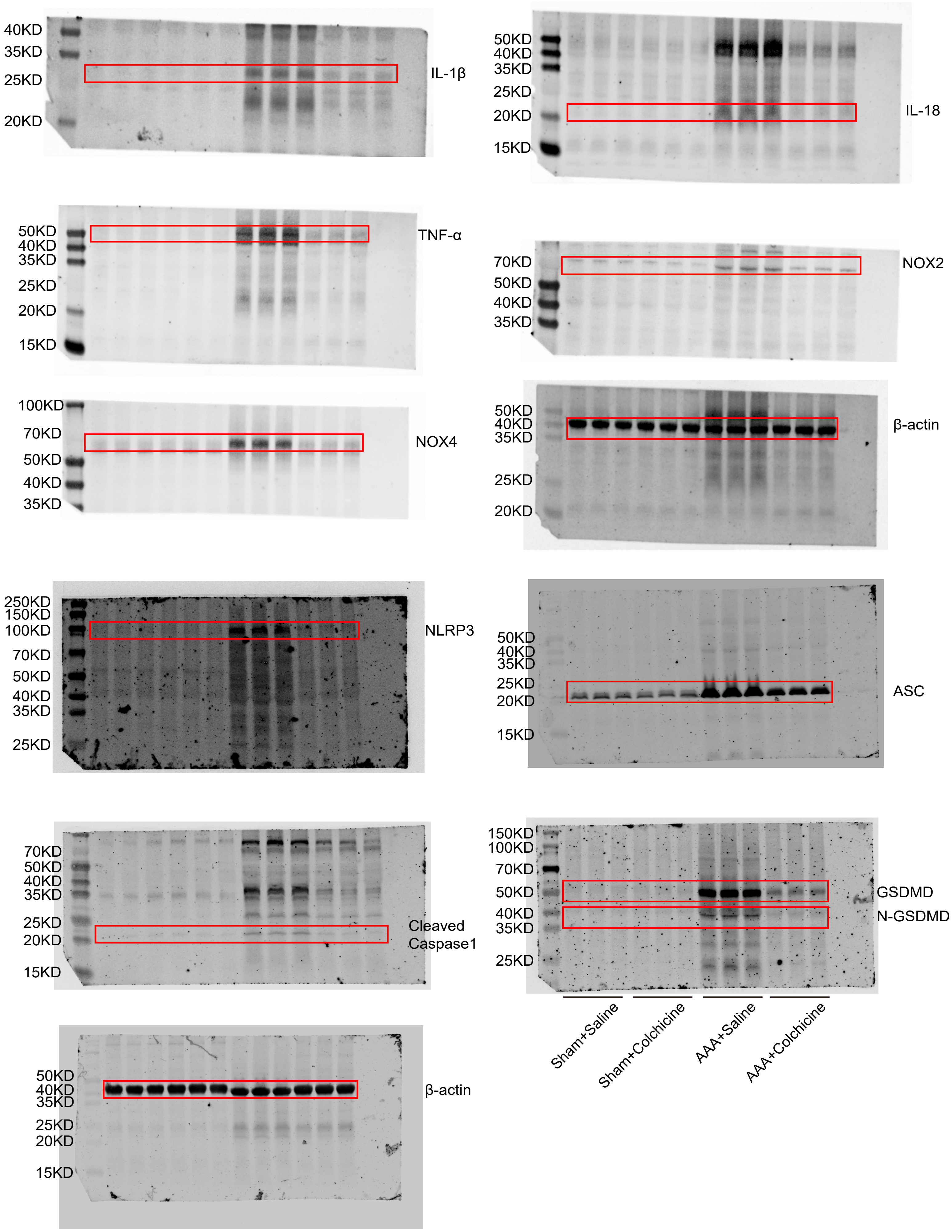

Figure 3 Supplementary material

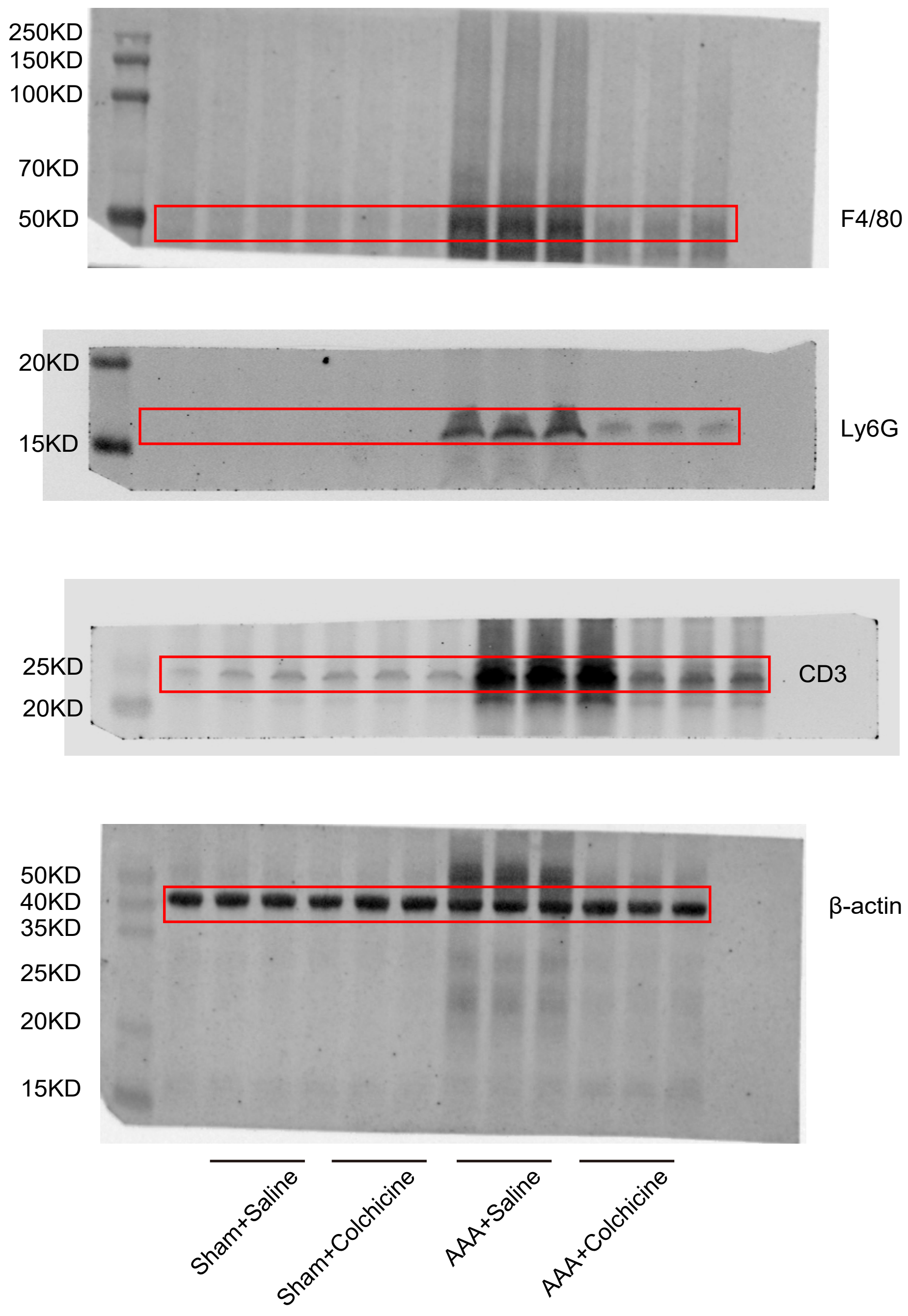

Figure 4 Supplementary material

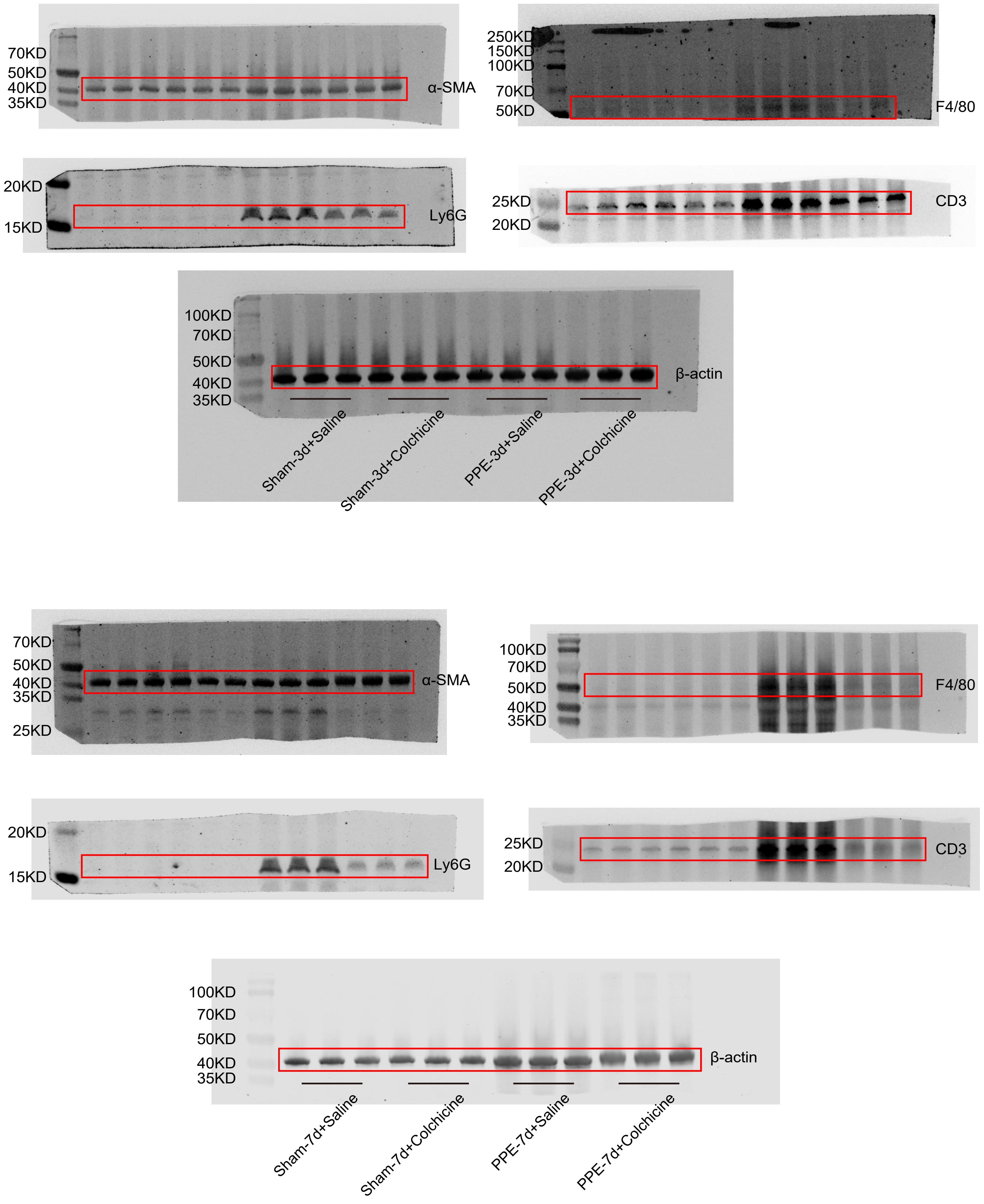

Figure 5 Supplementary material
